# Prevalence and determinants of nasal carriage of methicillin-resistant *Staphylococcus aureus* among internally displaced persons in Mogadishu, Somalia: A community based cross-sectional study

**DOI:** 10.1016/j.ijregi.2026.100851

**Published:** 2026-01-29

**Authors:** Shafie Abdulkadir Hassan, Mowlid Abdikarin Mohamed, Abdifetah Ibrahim Omar

**Affiliations:** 1 Center for Antimicrobial Resistance Research, Jamhuriya University of Science and Technology, Mogadishu, Somalia; 2 Department of Medical Laboratory Sciences, Faculty of Medicine and Health Sciences, Jamhuriya University of Science and Technology, Mogadishu, Somalia

**Keywords:** MRSA, Nasal carriage, Internally displaced persons (IDPs), Mogadishu, Somalia, Antimicrobial resistance

## Abstract

**Objectives:**

Internal displacement creates complex health challenges, particularly regarding the spread of antimicrobial resistance (AMR). In Somalia, internally displaced persons (IDPs) reside in overcrowded settlements with limited sanitation, creating conditions that favor the transmission of methicillin-resistant *Staphylococcus aureus* (MRSA). While clinical infections are reported, community-based data on asymptomatic carriage is scarce. This study investigated the prevalence and predictors of nasal MRSA carriage among IDPs in Mogadishu.

**Methods:**

A community-based cross-sectional study was conducted in the Daynile and Kahda districts of Mogadishu. A total of 428 IDPs were enrolled using systematic random sampling. Data on sociodemographics, housing, and medical history were collected via structured questionnaires. Anterior nasal swabs were cultured on Mannitol Salt Agar, and MRSA was confirmed using cefoxitin disk diffusion per Clinical & Laboratory Standards Institute (CLSI) guidelines. Multivariable logistic regression was used to identify independent predictors of carriage.

**Results:**

A total of 428 participants were enrolled (mean age 24.5 years). The overall prevalence of MRSA nasal carriage was 51.87% (222/428). In the multivariable analysis, the duration of displacement and household size were significant predictors of carriage. Recent arrival in the camp (1-15 months) was a strong risk factor (adjusted odds ratio = 2.92; 95% confidence interval: 1.64-5.19) compared to long-term residents. Additionally, smaller household sizes (1-4 members) showed higher odds of carriage (adjusted odds ratio = 2.08; 95% confidence interval: 1.23-3.51) compared to larger households, potentially reflecting specific housing vulnerabilities among newer, smaller family units.

**Conclusions:**

The burden of MRSA carriage is exceptionally high among displaced populations in Mogadishu. The findings suggest that recent displacement and living conditions associated with newer arrivals are critical drivers of transmission. Public health interventions should target recent arrivals with improved shelter and hygiene promotion to mitigate the spread of resistant pathogens.

## Highlights

• First community-based study of methicillin-resistant *Staphylococcus aureus* (MRSA) in Mogadishu internally displaced persons.
• MRSA nasal carriage prevalence was 51.9%.
• Recent displacement increased risk of carriage.
• Smaller households had higher MRSA odds.
• Targeted interventions needed to reduce antimicrobial resistance.

## Introduction

Antimicrobial resistance (AMR) is recognized by the World Health Organization as one of the top 10 global public health threats facing humanity [1,2]. Among the pathogens of concern, methicillin-resistant *Staphylococcus aureus* (MRSA) is significant due to its ability to cause a wide spectrum of diseases, ranging from skin and soft tissue infections to life-threatening sepsis [[3], [4], [5]]. While historically associated with hospitals, community-associated MRSA (CA-MRSA) has emerged as a dominant strain in low-resource settings [[6], [7], [8]].

The epidemiology of MRSA is intrinsically linked to environmental conditions [[9], [10], [11]]. Transmission occurs primarily through direct skin-to-skin contact and shared fomites [[12], [13], [14]]. Consequently, environments characterized by overcrowding and compromised hygiene act as incubators for transmission [15,16].

Somalia hosts one of the world’s largest internally displaced populations [17]. In Mogadishu, hundreds of thousands of internally displaced persons (IDPs) reside in the peripheral districts of Daynile and Kahda [18]. Residents often live in makeshift shelters offering poor ventilation and high density [19]. Although specific data on IDPs in Somalia is sparse, studies from refugee settings in other regions, such as Swiss refugee centers and camps in Ethiopia, have documented that the precarious nature of displacement significantly amplifies the risk of MRSA acquisition and clinical infection [11,20]. Furthermore, access to formal healthcare is limited, leading to reliance on informal drug sellers and unregulated antibiotic use [21,22].

Despite the biological plausibility for rapid MRSA transmission in these camps, community-based data remains limited. Previous Somali studies focused on clinical isolates from hospitals [23]. This study aims to bridge this gap by determining the prevalence of nasal MRSA carriage specifically among IDPs in Mogadishu and identifying modifiable risk factors.

## Methods

### Study design and setting

A descriptive cross-sectional study was conducted in the Daynile and Kahda districts of the Banadir Region (Mogadishu), which host the highest density of informal IDP settlements. The study was conducted from August to October, 2025.

### Study population

The population comprised individuals of all ages residing in the selected IDP camps.

• **Inclusion criteria:** Individuals residing in the camp for at least 3 months.
• **Exclusion criteria:** Active nasal pathology (defined as current rhinitis, nasal bleeding, or visible sores/abscesses), current intranasal medication use, or systemic antibiotic therapy within the last 48 hours.

### Ethical approval statement

The authors declare that this study was conducted in accordance with ethical standards and approved by the Jamhuriya Research Ethics Committee, Jamhuriya University of Science and Technology, Mogadishu, Somalia (Ref: JUREC00250/FMHS000132/062025). Written informed consent was obtained from all participants prior to enrollment.

### Sample size and sampling

The final sample size was 428 participants. The sample size was calculated using Cochran’s formula (n = Z²p(1-p)/d²). We assumed a prevalence (p) of MRSA carriage of 50% (as no similar local community study existed to provide a specific estimate), a 95% confidence level (Z = 1.96), and a 5% margin of error (d = 0.05). This yielded a base sample of 384, which was increased by 10% to account for non-response.

A systematic random sampling technique was employed. Four major settlements were selected. A sampling frame was created using household lists provided by camp leaders. We employed proportional allocation to determine the number of households to sample from each settlement based on their population size. A sampling interval (k) was used to select households (k = total households / sample size). The first household was selected using a lottery method (picking a number between 1 and k). One eligible participant was selected from each household using a lottery method.

### Data collection

Demographic and clinical data were collected using a pre-tested structured questionnaire. The questionnaire was interviewer-administered to ensure comprehension. Variables included sociodemographics (age, sex, duration of displacement), housing conditions (household size, sleeping rooms, water source, latrine type), and medical history (hospitalization, recent antibiotic use).

### Laboratory procedures

• **Specimen collection:** Sterile cotton-tipped swabs were collected from the anterior nares. Samples were placed in Amies transport medium, stored in a cool box at 4°C, and transported to the laboratory within 4 hours of collection.
• **Culture and identification:** Samples were inoculated on Mannitol Salt Agar and incubated at 37°C for 24-48 hours. *S. aureus* was identified by colony morphology, gram stain, catalase, and coagulase tests.
• **Antimicrobial susceptibility testing:** MRSA was identified using the cefoxitin (30 µg) disk diffusion method on Mueller-Hinton Agar per Clinical & Laboratory Standards Institute (CLSI) guidelines (M100, 33rd Edition) [24]. An inhibition zone of ≤21mm indicated methicillin resistance.

### Statistical analysis

Data were analyzed using SPSS Version 25.0. Frequencies and percentages were used for descriptive statistics. Bivariate analysis and multivariable logistic regression were performed to identify predictors of MRSA carriage. Results are reported as adjusted odds ratios (aOR) with 95% confidence intervals (CIs). Statistical significance was set at P<0.05

## Results

### Sociodemographic characteristics

A total of 428 IDPs participated in the study. The gender distribution was 53% (227/428) male and 47% (201/428) female. Most participants were in the age groups of 60-79 years (34.6%) and 45-59 years (26.2%). Regarding the duration of displacement, the largest group (43.9%) had resided in the camps for 1-15 months, indicating a population with many recent arrivals (Table 1).

**Table 1:** Sociodemographic characteristics of study participants (N = 428).

Variable	Frequency (n)	Percentage (%)
**Sex**		
Male	227	53.0
Female	201	47.0
**Age group**		
18–29 years	70	16.4
30–44 years	98	22.9
45–59 years	112	26.2
60–79 years	148	34.6
**Duration of displacement**		
1-15 months	188	43.9
16-30 months	85	19.9
31-45 months	79	18.5
≥46 months	76	17.8

### Medical and housing history

More than half of the participants (52.1%) had been hospitalized in the last 12 months. Antibiotic usage was high, with 49.3% reporting antibiotic use in the past 3 months and 50.2% obtaining antibiotics over-the-counter (OTC). Housing conditions revealed that 34.3% lived in small households (1-4 members), while 39.5% lived in households of 5-8 members. Most participants (62.1%) reported having 3-5 sleeping rooms. Water sources varied, with 35.3% using piped water and 34.8% relying on tankers (Table 2, Table 3).

**Table 2:** Medical history and antibiotic use (N = 428).

Variable	Frequency (n)	Percentage (%)
**Hospitalized in last 12 months**		
Yes	223	52.1
No	205	47.9
**Antibiotic use in past 3 months**		
Yes	211	49.3
No	217	50.7
**Obtained antibiotics over-the-counter**		
Yes	215	50.2
No	213	49.8
**Shared antibiotics with others**		
Yes	199	46.5
No	229	53.5

**Table 3:** Household and living conditions (N = 428).

Variable	Frequency (n)	Percentage (%)
**Household size**		
1-4 members	147	34.3
5-8 members	169	39.5
9-11 members	112	26.2
**Number of sleeping rooms**		
1-2 rooms	162	37.9
3-5 rooms	266	62.1
**Source of water**		
Piped water	151	35.3
Tanker (Truck)	149	34.8
Well	128	29.9
**Latrine type**		
Flush toilet	152	35.5
Pit latrine	138	32.2
Open defecation	138	32.2

### Prevalence of MRSA carriage

**Figure 1:**
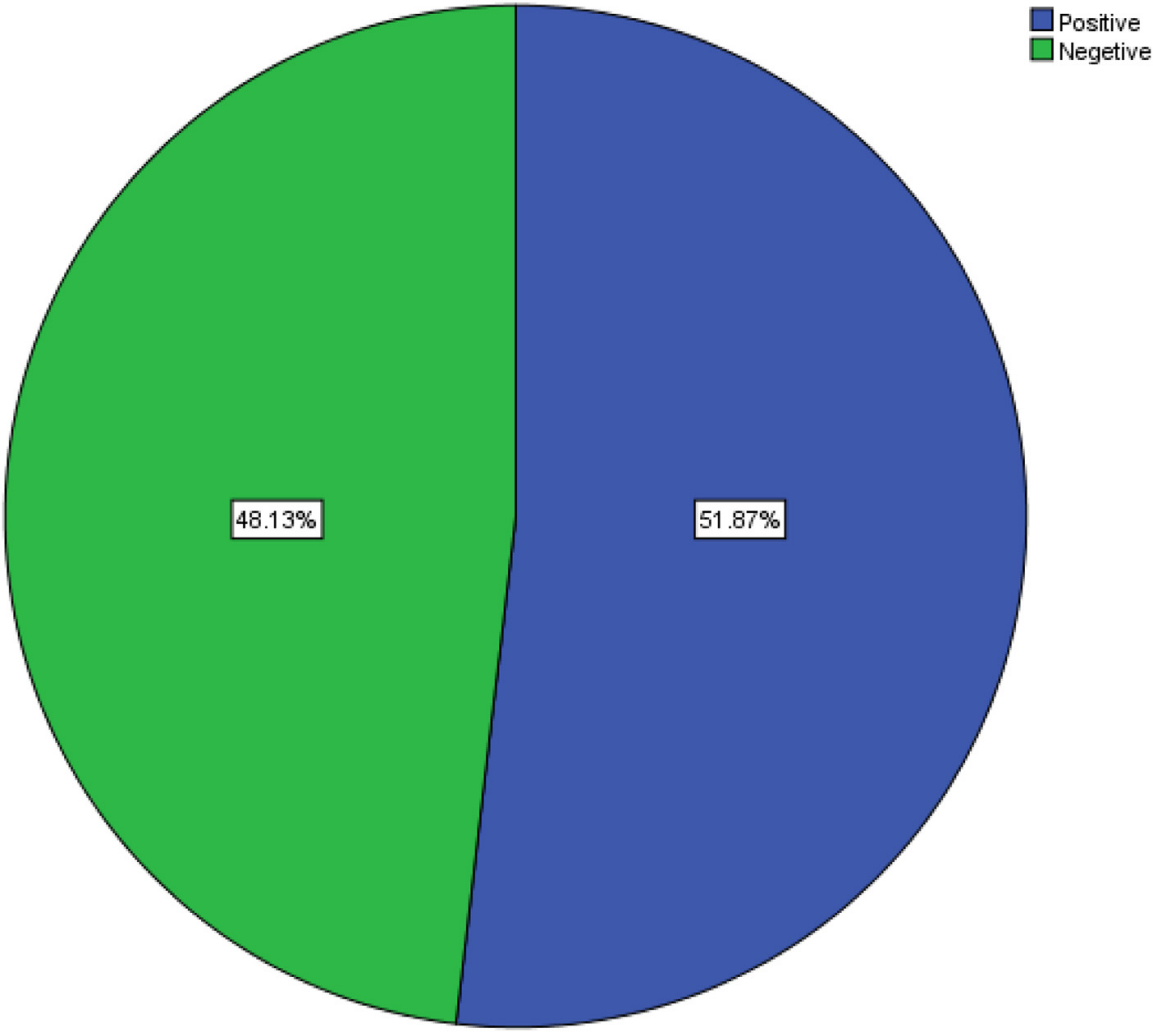
Prevalence of methicillin-resistant *Staphylococcus aureus* carriage among internally displaced persons in Mogadishu, Somalia.

Laboratory analysis of nasal swabs from 428 study participants revealed *Staphylococcus aureus* colonization in 60.5% (259/428) of individuals. Of these *S. aureus*-positive individuals, 222 were colonized with MRSA. Consequently, the proportion of MRSA among *S. aureus* isolates was 85.7% (222/259), and the overall prevalence of MRSA nasal carriage in the study population was 51.9% (222/428) (Figure 1).

### Determinants of MRSA carriage

The multivariable logistic regression analysis identified specific risk factors independently associated with MRSA carriage. Duration of displacement emerged as a critical predictor, where participants who had recently arrived in the camp (1-15 months) demonstrated significantly higher odds of colonization compared to long-term residents (aOR = 2.919; 95% CI: 1.641-5.194; *P* = 0.001). Furthermore, household size was found to be a significant determinant; surprisingly, individuals living in smaller households (1-4 members) were approximately twice as likely to carry MRSA compared to those in larger family units (aOR = 2.076; 95% CI: 1.229-3.508; *P* = 0.013). In contrast, after adjusting for confounders, other variables, such as sex, age, history of hospitalization, recent antibiotic use, and the type of water source or latrine were not found to be statistically significant predictors of carriage (Table 4).

**Table 4:** Bivariate and multivariable logistic regression analysis of factors associated with MRSA nasal carriage.

Variable	MRSA positive	MRSA negative	Crude OR (95% CI)	Adjusted OR (95% CI)	*P*-value
**Sex**					
Male	126	101	1.36 (0.93-1.99)	1.41 (0.94-2.11)	0.097
Female	96	105	1.00	1.00	
**Age**					
18-29 years	43	27	1.59 (0.85-2.97)	1.80 (0.91-3.57)	
30-44 years	49	49	1.84 (1.00-3.38)	1.62 (0.84-3.09)	0.342
45-59 years	52	60	1.43 (0.80-2.55)	1.34 (0.71-2.50)	
60-79 years	78	70	1.00	1.00	
**Duration of displacement**					
1-15 months	105	83	2.58 (1.51-4.40)	**2.92 (1.64-5.19)**	**0.001**^a^
16-30 months	28	57	1.11 (0.66-1.89)	1.31 (0.74-2.33)	
31-45 months	42	37	0.78 (0.45-1.35)	0.83 (0.46-1.52)	
≥46 months	47	29	1.00	1.00	
**Hospitalized (last 12 months)**					
Yes	115	108	1.03 (0.70-1.50)	0.94 (0.62-1.41)	0.750
No	107	98	1.00	1.00	
**Antibiotic use (past 3 months)**					
Yes	105	106	1.18 (0.81-1.73)	0.85 (0.57-1.28)	0.441
No	117	100	1.00	1.00	
**Household size**					
1-4 members	64	83	1.79 (1.09-2.95)	**2.08 (1.23-3.51)**	**0.013**^a^
5-8 members	93	76	1.13 (0.70-1.83)	1.20 (0.73-1.99)	
9-11 members	65	47	1.00	1.00	
**Water source**					
Piped water	77	74	1.09 (0.69-1.71)	1.10 (0.68-1.78)	
Well	66	62	1.06 (0.66-1.70)	1.13 (0.68-1.86)	0.884
Tanker	79	70	1.00	1.00	
**Latrine type**					
Flush toilet	72	80	1.44 (0.91-2.30)	1.46 (0.89-2.37)	
Pit latrine	72	66	1.19 (0.74-1.92)	1.17 (0.71-1.93)	0.316
Open defecation	78	60	1.00	1.00	

a Statistically significant at p < 0.05. CI, confidence interval; MRSA, methicillin-resistant *Staphylococcus aureus*; OR, odds ratio*.*

## Discussion

This study represents a critical community-based assessment of MRSA nasal carriage among IDPs in Mogadishu. The results reveal an alarmingly high prevalence of MRSA carriage at 51.87%. This figure is significantly higher than rates typically reported in non-displaced community settings globally and exceeds findings from many other refugee settings [23,[25], [26], [27]]. This suggests that the IDP settlements in Mogadishu may act as high-intensity reservoirs for resistant pathogens.

The strongest predictor of MRSA carriage was the duration of displacement. Individuals who had arrived recently (1-15 months) had nearly three times the odds of carriage compared to long-term residents. This “new arrival effect” may be attributed to the acute stressors of displacement. Recent arrivals often inhabit the most precarious shelters, lack established hygiene routines, and may have lower immunity due to the stress of migration and malnutrition. Conversely, long-term residents may have acquired better housing or adapted hygienically to the environment over time. Consistent with our findings, studies conducted in refugee settings have identified displacement-related living environments as a key risk factor for adverse MRSA outcomes. In particular, residence in refugee centers has been associated with increased MRSA persistence and treatment failure, underscoring the role of crowded and resource-limited settings in sustaining MRSA carriage [20].

Contrary to the traditional hypothesis that larger families facilitate overcrowding and spread, our study found that smaller households (1-4 members) were at higher risk. In the context of Mogadishu’s IDP camps, small household size might be a proxy for socioeconomic vulnerability. Small family units may represent fragmented families or recent arrivals living in smaller, less ventilated shelters with poorer sanitation infrastructure compared to larger, established families who may occupy larger compounds [28]. Further qualitative research is needed to understand this dynamic.

While recent antibiotic use was high (49.3%), it did not remain a statistically significant predictor in the multivariable model (P=0.44). However, the high descriptive rate of OTC antibiotic acquisition (50.2%) points to a widespread lack of antimicrobial stewardship, which likely contributes to the high baseline community resistance rates observed in the overall prevalence.

### Limitations

This study has limitations. As a cross-sectional design, it establishes association but not causality. The high prevalence rate warrants molecular characterization (e.g., *mecA* gene polymerase chain reaction) to distinguish between CA-MRSA and HA-MRSA clones, which was not performed due to resource constraints. Additionally, data on antibiotic use was self-reported, potentially introducing recall bias.

## Conclusion

The prevalence of MRSA nasal carriage among IDPs in Mogadishu is critical (51.87%). The study identifies recent arrivals (1-15 months duration) and smaller household units as the most vulnerable groups. To address this, health partners must implement targeted screening and decolonization for new entrants, urgently upgrade shelter sanitation, and enforce strict antimicrobial stewardship to curb unregulated antibiotic sales.

## Declaration of competing interest

The authors have no competing interests to declare.
